# MicroRNA as a Therapeutic Target in Cardiac Remodeling

**DOI:** 10.1155/2017/1278436

**Published:** 2017-09-28

**Authors:** Chao Chen, Murugavel Ponnusamy, Cuiyun Liu, Jinning Gao, Kun Wang, Peifeng Li

**Affiliations:** Center for Developmental Cardiology, Institute for Translational Medicine, Qingdao University, Dengzhou Road 38, Qingdao 266021, China

## Abstract

MicroRNAs (miRNAs) are small RNA molecules that contain 18–25 nucleotides. The alterations in their expression level play crucial role in the development of many disorders including heart diseases. Myocardial remodeling is the final pathological consequence of a variety of myocardial diseases. miRNAs have central role in regulating pathogenesis of myocardial remodeling by modulating cardiac hypertrophy, cardiomyocytes injury, cardiac fibrosis, angiogenesis, and inflammatory response through multiple mechanisms. The balancing and tight regulation of different miRNAs is a key to drive the cellular events towards functional recovery and any fall in this leads to detrimental effect on cardiac function following various insults. In this review, we discuss the impact of alterations of miRNAs expression on cardiac hypertrophy, cardiomyocytes injury, cardiac fibrosis, angiogenesis, and inflammatory response. We have also described the targets (receptors, signaling molecules, transcription factors, etc.) of miRNAs on which they act to promote or attenuate cardiac remodeling processes in different type cells of cardiac tissues.

## 1. Introduction

**Figure 1:**
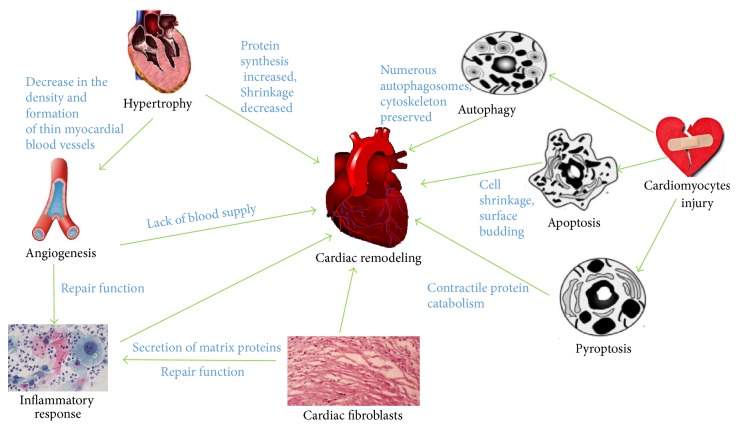
The risk factors for cardiovascular diseases and myocardial remodeling.

Myocardial remodeling is one of the major alterations of architecture of the heart that causes the hemodynamic imbalance under pathological stimuli and biomechanical stresses of external stimuli. Myocardial remodeling is a detrimental cardiac problem due to the reason that it is associated with nearly all kinds of heart diseases. The myocardial remodeling process includes physiological changes in structure, dimensions, mass, shape, and functions of the heart as well as cardiac cells. The major contributors in the pathological of myocardial remodeling are cardiac hypertrophy, cardiomyocytes injury, cardiac fibrosis, angiogenesis, and inflammatory response. It is well known that a significant loss of cardiomyocytes due to various insults and slower rate of cardiomyocyte regeneration is the initiative event for the myocardial remodeling in human as well as in animal models [1]. The cardiac inflammation following the myocardial infarction and ischemia reperfusion injury is the primary driving force of delayed cardiac regeneration and pathological cardiac remodeling. The production of extracellular matrix components (ECM) is essential to provide mechanical support to the regenerating heart after cardiac injury. However, the dynamic change in ECM production forces the heart tissue to maladaptive repair, prolonged inflammatory response, and loss of cardiomyocytes, which ultimately leads to cardiac remodeling. The relationship between the risk factors for cardiovascular diseases and myocardial remodeling is shown in Figure 1.

miRNAs are transcripts of noncoding region of the gene. The miRNA era began in 1993 when Lee et al. found the first microRNA in nematode. This field of research has made a tremendous progress in recent years and now it is realized that miRNAs have unavoidable role in both physiological as well as pathological processes [2]. The precursor molecules of miRNAs are called primary miRNAs (pre-miRNAs), which are derived as long fragments in the nucleus by RNA polymerase II. The pre-miRNAs are translocated to the cytoplasm and subsequently processed by different molecular machineries including RNase III-type endonuclease and Dicer, to form a mature double-stranded miRNAs. In maturation process, the pre-miRNAs lose their terminal base pairs and their hairpin structure in order to generate a mature miRNA. The single-stranded form is a fully active mature miRNA, which binds with RNA-induced silencing complexes (RISCs) and specifically binding with its target genes. These binding silences target gene activity at posttranscriptional level [3, 4]. Many experimental and clinical studies reveal that the expression of miR is highly altered in various cardiovascular problems and their alterations are linked with modulation of activity and expression of key components of cardiomyocyte growth, survival, and death. Thus, miRNAs are considered as a central part of the development of various cardiac disorders, which subsequently leads to cardiac remodeling and progression to heart failure. In this review, we discuss the impact of alterations of miR expression on the process of pathological cardiac remodeling. For the clear illustration, we divide the adverse cardiac remodeling into the following components: cardiac hypertrophy, cardiac fibrosis, myocardial cell injury, and angiogenesis, which are closely related to the progression of cardiac remodeling and heart failure.

## 2. miRNA and Cardiac Hypertrophy

Cardiac hypertrophy is one of the major responses to various physiological and pathological stress stimuli in the heart. The major feature of hypertrophic response is an increase in the thickness of the ventricular wall, cardiac dilatation, and heart failure. As the maladaptive hypertrophic response consequently leads to various cardiac problems, many scientific works endeavor to figure out the underlying molecular mechanism of pathological hypertrophy. However, the molecular mechanisms associated with cardiac hypertrophy remain largely unknown [5, 6]. miRNAs play an important role in regulating the cardiac hypertrophic processes. They either negatively or positively modulate hypertrophic signaling and thus indirectly influence the hypertrophy associated cardiac remodeling.

MiR-1 is one of the key regulators of pathological cardiac hypertrophy by targeting multiple signaling molecules in human as well as in experimental animal models. MiR-1 protects heart structure and functions against cardiac hypertrophic responses by directly targeting and inhibiting translation of many signaling molecules including eukaryotic initiation factor 4E (Eif4e), Mef2a, Gata4, and histone deacetylase 6 (HDAC6) [7–9]. MiR-1 also attenuates calcium signaling dependent cardiac hypertrophic response by negatively regulating calmodulin (CAM), one of the key components of calcium signaling, which contributes to the progression of pathological hypotrophy in the heart [7]. The increased circulatory level of heart specific fat binding protein 2 (FABP3) is an indicator of heart enlargement and hypertrophy in patients and miR-1 can directly target FABP3 in cardiomyocytes. A study found that there is an inverse correlation between the expression of miR-1 in myocardial tissue and FABP3 level in circulation [10]. Another research group found that miR-1 inhibits fibulin-2 (Fbln2) expression and thereby it abolishes activation of TGF*β* signaling and extracellular matrix remodeling in hypertrophic heart [11, 12]. MiR-133 is one of the abundantly expressed antihypertrophic miR in both animal and human myocardial tissues. MiR-133 blocks hyperthyroidism induced cardiac hypertrophy by suppressing the expression of type 1 angiotensin II receptor (AT1R) [13]. Carè et al. (2007) observed that miR-133a attenuates cardiac remodeling by regulating Akt and its downstream signaling molecules such as Cdc42, Rho-A, and Nelf-A/WHSC2 [14]. MiR-10a [15] and miR-497 [16] exert their antihypertrophic effect by blocking translation of Tbx2 and Sirt4, respectively. MiR-223 directly targets cardiac troponin I-interacting kinase (TNNI3K) and inhibits troponin I (cTnI) phosphorylation in cardiomyocytes, which promotes contractility and accumulation of intracellular Ca^2+^ [17]. MiR-455 is an important miR in the developing heart that targets calreticulin to prevent pressure overload induced hypertrophy [18]. MiR-378 suppresses the expression and activities of many growth promoting receptors and signaling pathways, including insulin-like growth factor receptor 1 (IGFR1), growth factor receptor bound protein 2 (Grb2), kinase suppressor of Ras 1 (Ksr1), Ras activity, PI3K-Akt pathway, Mapk1-MAPK signaling, and Raf1-MEK1-ERK1/2 pathway [19, 20] This inhibition is associated with reduction of hypertrophic response in cardiac tissue. MiR-145 showed its negative effect in isoproterenol-induced cellular hypertrophy via regulation of the expression of GATA binding protein 6 (GATA6) [21, 22]. MiR-155 alleviates cardiac hypertrophy and improves heart function by repressing the expression of angiotensin II receptor type 1 (AGTR1) and suppression of its downstream calcium signaling pathways [23]. MiR-212/132 family [24] and miR-23a [25] target Foxo3 transcription factor to mitigate the hypertrophic signal in cardiomyocytes. MiR-29a-3p inhibits ET-1-induced hypertrophic response in cardiomyocytes by directly targeting 3′ UTR of NFATc4 [26].

In opposite, many miRNAs play prohypertrophic role in the heart. For instance, miR-208a is a well expressed miR in the heart and it is highly associated with development of cardiac hypertrophy and fibrosis. Hyperthyroidism induces cardiac hypertrophy by promoting the expression of angiotensin type 1 receptor (AT1R). The stimulation of *α*-MHC mediated expression of miR-208a promotes the expression of AT1R in hyperthyroid condition [27]. Another study found that miR-208 regulates the translation of thyroid hormone receptor associated protein 1 (THRAP1) and leads to hyperthyroidism under stress condition. This event consequently enhances *β*-MHC expression and further aggravates cardiac hypertrophy [28]. MiR-124 effectively represses AngII-induced neonatal cardiomyocytes hypertrophy by blocking the expression of calreticulin (CRT) and glucose-regulated protein (Grp78), an endoplasmic reticulum (ER) stress marker [29]. Several miRNAs reduce the expression of antihypertrophic factors and indirectly contribute to elevation of pathological hypertrophy. MiR-297 negatively regulates the expression of Sigma-1 receptor (Sig-1R) and activates ER stress signaling [30], while miR-17-3p targets metallopeptidase inhibitor 3 (TIMP3), a negative regulator of PTEN-Akt pathway, and resulting in cardiomyocyte hypertrophy [31]. MiR-21 targets suppressors of hypertrophic response by inhibiting translation of SH3 domain-containing protein 2 (SORBS2), PDZ, and LIM domain 5 (PDLIM5) [32]. MiR-155 acts as a prohypertrophic factor by abolishing the expression of Mef2A and Jarid2 in cardiomyocytes and inhibition of miR-155 improves the cardiac function from hypertrophy induced progressive heart failure [33]. MiR-22 acts as a prohypertrophic factor by targeting PTEN, Sirtuin 1 (Sirt1), and histone deacetylase 4 (Hdac4), which are negative regulators of hypertrophic signaling [25, 34, 35]. Interestingly, miR-23a has mutual regulatory role with lysophosphatidic acid (LPA) receptor. LPA can upregulate miR-23a expression by activation of LPA3 dependent PI3K/Akt pathway [36].

**Figure 2:**
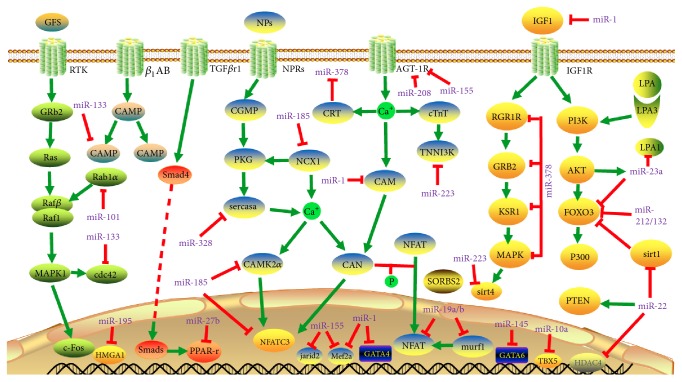
*MicroRNAs (miRNAs) in cardiac hypertrophic pathways*. Arrows colored in red indicate the functions of depression; arrows colored in green indicate the functions of activation. Abbreviations for mRNAs: AGTR1: angiotensin II receptor type 1; Camk2d: calcium/calmodulin-dependent protein kinase II delta; Cdc42: cell division cycle 42; c-Fos: proto-oncogene protein; CRT: calreticulin; Foxo3: Forkhead box O3; Gata4: GATA binding protein 4; GATA6: GATA binding protein 6; Grb2: growth factor receptor bound protein 2; Hdac4: histone deacetylase 4; HMGA1: high mobility group AT-hook 1; NFAT: nuclear factor of activated T cells; IGF-1: insulin-like growth factor 1; IGF-1R: insulin-like growth factor 1 receptor; Jarid2: jumonji and AT-rich interaction domain-containing protein 2; Ksr1: kinase suppressor of ras 1; LPA1: lysophosphatidic acid receptor 1; Mapk1: mitogen-activated protein kinase 1; Murf1: tripartite motif-containing 63; NCX1: sodium/calcium exchanger 1; Nfatc3: nuclear factor of activated T cells, cytoplasmic, calcineurin dependent 3; Ppar*γ*: peroxisome proliferator-activated receptor *γ*; PTEN: phosphatase and tensin homolog; p300: E1A binding protein p300; Rab1A: Ras-related protein Rab 1a; Serca2a: Sarco/endoplasmic reticulum Ca^2+^-ATPase 2a; SIRT1: Sirtuin 1; SMAD4: SMAD family member 4; SORBS2: SH3 domain-containing protein 2; TGF-*β*R1: transforming growth factor *β* receptor 1; TNNI3K: troponin I type 3 interacting kinase; cAMP: cyclic adenosine monophosphate; cGMP: cyclic guanosine monophosphate; PKG: cGMP-dependent protein kinase; CAM: calmodulin; CTnT: cardiac troponin T; NPs: natriuretic peptides.

In experimental cardiac hypertrophy, MiR-195 downregulates the translation of HMGA1 by targeting its 3′ UTR and this inhibition promotes hypertrophic response [37, 38]. Atrogin 1 and muscle RING finger protein 1 (Murf1) are antihypertrophic factors and their expression is downregulated by miR-19a/b family. miR-19b mediates hypertrophic process by activating the PI3K and calcineurin/nuclear factor of activated T cells (NFAT) signaling, which are prohypertrophic pathway that changes phonotype of cardiomyocytes. Interestingly, miR-19b also has the capability to promote cell survival by attenuating the upregulation of NFAT target gene encoding *α*-crystallin-B and repression of the proapoptotic gene Bim (Bcl-2-interacting mediator of cell death) caused by endoplasmic reticulum (ER) stress [39]. A study in experimental model of hypertrophy found that miR-27b blocks the expression of peroxisome proliferator-activated receptor *γ* (PPAR-*γ*), which can relieve cardiac hypertrophy mediated cardiac dysfunction and heart failure. In pressure overload model of hypertrophy, it was confirmed that antagonists of miR-27b can restore the expression of PPAR-*γ* and alleviate heart dysfunction in those animals. Surprisingly, transforming growth factor *β*1 (TGF*β*1), a hypertrophy stimulant, can regulate the expression of miR-27b. [40]. Sarcoplasmic reticulum Ca2+ ATPase (SERCA2a) is a key calcium transporter responsible for the Ca^2+^ reuptake, which is suppressed by miR-328 during hypertrophic response in cardiomyocytes as well as in hearts of mouse. This leads to upregulation of the calcineurin/NFAT signaling and cardiac dysfunction [41]. Several experimental studies also found that the suppression of expression of hypertrophy associated miRNAs using antagonists (antagomir) attenuates pathological remodeling in the heart. miR-185 favours hypertrophic response by upregulating multiple genes in calcium signaling pathways, including Ca^2+^/CaM dependent protein kinase II*δ* (Camk2d), sodium/calcium exchanger 1 (Ncx1), nuclear factor of activated T cells, and cytoplasmic calcineurin dependent 3 (Nfatc3) and downregulation of miR-185 can alleviate hypertrophy induced pathological changes in the heart [42]. Similarly, antagonist of miR-101 attenuates experimental cardiac hypertrophy by target the expression of ras-related protein-1A (Rab1A) [43]. The antimir of miR-208a attenuates cardiac remodeling by upregulation of expression Myh7 in pressure overload induced heart failure in rats [44]. Together, these studies reveal that the balance in the expression of certain miRNAs is essential to control the hypertrophic response in cardiomyocytes and preservation of cardiac structure and function. The known microRNAs (miRNAs) in cardiac hypertrophic and their target genes are summarized in Figure 2 and Table 1.

## 3. miRNA and Fibrosis

Cardiac fibroblast is the most abundant cell type in the heart that comprises about two-thirds of the total number of cardiac cell types. The transient activation and proliferation of cardiac fibroblasts after cardiac injury are vital to maintain cardiac integrity and function. However, the persistence of fibroblast activation process accumulates myofibroblast phenotype in the cardiac tissue and that causes adverse myocardial remodeling and cardiac dysfunction. The relentless availability of growth factors, abundant expression of growth factor receptors, and accumulation of cytoplasmic matrix proteins play a fundamental role in the activation of cardiac fibroblast and fibrotic processes [45, 46]. The other factors such as reactive oxygen species (ROS), inflammatory cytokines and chemokines, and mast cell-derived proteases also play crucial role in the development of myocardial fibrosis (Figure 2). Many miRNAs have influence on fibrogenesis either positively or negatively by modulating the expression/activities of fibrotic signaling molecules in the cardiac tissue.

Transforming growth factor *β* (TGF*β*) is a multifunctional cytokine that plays a central role in the development and progression of fibrosis in many tissues including heart. The activation TGF*β* dependent signaling pathway and relentless expression of TGF*β* signaling components play crucial role in the expression of extracellular matrix components (ECM) and aggravation of fibrotic response, which consequently leads to ventricular remodeling in the heart. Several miRNAs exert their antifibrotic activity by directly antagonizing the expression and/or activity of TGF*β* signaling components in cardiac myofibroblasts. MiR-101 blocks excessive production of matrix proteins and proliferation of activated fibroblasts by suppressing expression of TGF*β*RI and c-Fos. In experimental model, overexpression of miR-101 alleviates deterioration of cardiac performance caused by fibrotic process [47, 48]. MiR-122 can control the expression of TGF-*β*1, but its expression is downregulated in patients and leads to severe myocardial fibrosis [49]. MiR-24 inhibits TGF*β*-smad2/3 signaling mediated differentiation and migration of cardiac fibroblasts by inhibiting furin, a regulatory protein involved in latent TGF*β* activation process. A synthetic precursor of miR-24 can improve heart function [50]. Similarly, MiR-98 and miR-19a-3p/19b-3p inhibit fibrogenesis by blocking the expression of TGF*β*RI and TGF*β*RII, respectively. These receptors are integral part of ECM production, cardiac fibrosis, and ventricular thickening [51, 52]. The miR-15 family is one of the most important miRNAs involved in the regulation of fibrosis. It encodes six miRNAs (miR-15a, miR-15b, miR-16, miR-195, miR-497, and miR-322). The expression of multiple miR-15 family members targets TGF-*β*R1, p38, SMAD3, SMAD7, and endoglin to inhibits the TGF-*β*-pathway. However, the family of miR-15 has negative effect on cardiomyocyte proliferation [53, 54]. Likewise, miR-378 attenuates TGF/Smad/Ras signaling by suppressing the expression of several profibrotic transcription factors such as AP1 transcription factors, c-Fos, and c-Jun in cardiac fibroblasts. However, this miRNA expression is downregulated in cardiac tissue during fibrotic process by an unknown mechanism [55]. Several miRNAs play profibrotic role by upregulating the expression of TGF*β* signaling molecules. miR-21 is highly expressed by cardiac fibroblasts in postmyocardial injury condition and its expression accelerates fibrotic process by blocking the expression of TGF*β*RIII, which is a negative regulator of TGF*β*1-Smad3 signaling [56]. miR-21 also favours fibrogenic process by inhibiting the expression of sprouty homolog 1 (Spry1), a molecule required for controlling fibroblast growth factor secretion and inhibition of ERK–MAP kinase activity. miR-155 increases TGF*β*1–Smad2 signaling pathway by elevating the level of Smad 2 and resulting in cardiac fibrosis [57]. TGF*β*1 induces expression of miR-22, which increases the level of Smad4, which is an essential component of Smad complex (Smad2/3/4) that facilitates the translocation to nucleus and induction of downstream genes [58].

Connective tissue growth factor (CTGF) is a key molecule involved in the development and progression of fibrosis by promoting the synthesis of ECM proteins including collagen. miR-133 and miR-30 downregulate CTGF expression and attenuate cardiac fibrosis [59]. miR-26a also attenuates fibrotic process by directly targeting translation of CTGF genes and expression of collagen type I. Interestingly, NF-kB and miR-26a reciprocally regulate each other in the fibrotic heart. The increased expression of NF-kB diminishes levels of miR-26a, while overexpression of miR-26a diminishes NF-kB activation [60]. A recent study found that miR29 acts as an antifibrotic factor by accelerating the expression of multiple extracellular matrix genes including elastin [61], fibrillin 1 (Fbn1), collagen type I, *α* 1 and 2 (Col1*α*1, Col1*α*2), collagen type III, *α*1 (Col3*α*1), and fibronectin (FBN) [62, 63]. Another research group also confirmed the antifibrotic effects of miR-29b in angiotensin II induced cardiac fibrosis model [64]. MiR-146a targets VEGF expression and suppresses VEGF dependent proliferation and expansion myofibroblasts [65]. The other miRNAs such as miR-142-3p and miR-433 target high mobility group box 1 (HMGB1), AZIN1, and JNK1, respectively, to ameliorate cardiac fibrosis [66, 67].

**Figure 3:**
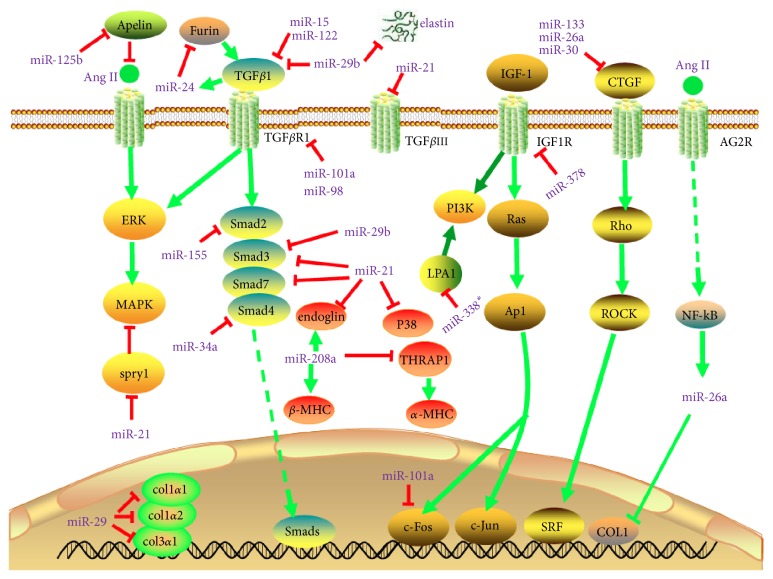
*MicroRNAs (miRNAs) in cardiac fibrosis pathways*. Arrows colored in red indicate the functions of depression; arrows colored in green indicate the functions of activation. Abbreviations for mRNAs: *β*-MHC: beta myosin heavy chain; COL1: collagen, type 1; Col1*α*1: collagen, type 1 *α* 1; Col1*α*2: collagen, type 1 *α* 2; Col3*α*1: collagen, type 3 *α* 1; CTGF: connective tissue growth factor; IGF-1: insulin-like growth factor 1; IGF-1R: insulin-like growth factor 1 receptor; p38: Tumor protein p38; ROCK1: Rho associated coiled-coil containing protein kinase 1; SMAD3: SMAD family member 3; SMAD2: SMAD family member 2; SMAD7: SMAD family member 7; Spry1: sprouty homolog 1; TGF-*β*1: transforming growth factor *β* 1; TGF-*β*R1: transforming growth factor *β* receptor 1; TGF*β*RIII: transforming growth factor *β* receptor III; THRAP1: mediator complex subunit 13; ERK: extracellular regulated protein kinases; MAPK: mitogen-activated protein kinase; AG2R: anterior gradient-2.

Several miRNAs positively modulate myocardial fibrosis by directly upregulating the expression of profibrotic genes or indirectly through suppressing the expression of antifibrotic signaling molecules, which inhibit fibrotic process by either negatively regulating profibrotic factors activity or expression. miR-208 is a cardiac-specific miR whose upregulation is closely related to the development of cardiac fibrosis. miR-208 induces myocardial fibrosis by upregulating the expression of endoglin, a coreceptor of TGF and *β*-myosin heavy chain (*β*-MHC) [68, 69]. A molecular study found that miR-208 specifically contributes to stress dependent expression of *β*-MHC and cardiac fibrosis. This is partly due to suppression of thyroid hormone receptor associated protein 1 (THRAP1), a coregulator of thyroid receptor, which can positively modulate *α*-MHC gene expression and suppression of *β*-MHC in cardiac tissue [28]. Interestingly, miR-22 acts as a downstream of TGF*β*1 signaling. TGF*β*1 induces expression of miR-22, which increases the level of Smad4, an essential component of Smad complex (Smad2/3/4) that facilitates the translocation to nucleus and induction of downstream genes [58]. miR-499 plays a crucial role in the development of myocardial hypertrophy and fibrosis by targeting many intracellular signaling molecules and transcription factors including Akt, MAPKs, Egr1, Egr2, and Fos or promoting Myh7 and Acta1 expressions [68]. miR-125b acts as a profibrotic molecule by targeting apelin, which is one of the key suppressors of cardiovascular remodeling that inhibits TGF*β* mediated collagen production and Ang II induced plasminogen activator inhibitor type I (PAI-1) [70, 71]. Collectively, these studies reveal that miRNAs play unavoidable role in both controlling and activating fibrogenic processes and cardiac remodeling. The balancing of antifibrotic miRNAs and profibrotic miRNAs is crucial to determine the fibroblast activation, acceleration of fibrotic signaling, accumulation of ECM components, and fibrotic processes. The known miRNAs in cardiac fibrosis and their target genes are summarized in Figure 3 and Table 2.

## 4. miRNA and Cardiomyocytes Injury

The loss of cardiomyocytes due to injury and their low regenerative capability in the adult heart is the early event responsible for the ventricular dysfunction, fibrosis, and progressive heart failure. Many studies found that chronic cardiac remodeling is highly associated with increasing cardiomyocyte cell death. The damage and/or loss of cardiomyocytes occur in various cardiovascular problems including myocardial infarction, ischemia/reperfusion injury, cardiomyopathy, and cardiac toxicity. However, the mode of cell death mainly depends on the type of insult to the heart. In the last few years, numerous miRNAs have been identified as regulators of cardiomyocytes injury due to their role in apoptosis, autophagy, and inflammatory response. In this part, we summarize the influence of miRNAs on different mode of cell death in cardiomyocytes.

### 4.1. miRNA and Apoptosis

Apoptosis is the major form of cardiomyocyte death in the failing heart. In cardiomyocytes, apoptosis can be activated by both intrinsic pathway (mediated by mitochondria) and extrinsic pathway (mediated by plasma membrane death receptors). However, these two pathways converge into mitochondria to induce the release of mitochondrial apoptogenic molecules and activation of caspase cascades. The activation of apoptosis is a complex event, which is tuned by highly regulated interaction of proapoptotic molecules. Many cell signaling pathways including the phosphatidylinositol 3-kinase (PI3-K)/Akt pathway, p53 pathway, activators of transcription (STAT) pathway, mitogen-activated protein kinase (MAPK) pathway, and stress-activated protein kinase (SAPK) signaling pathway contribute to the activation and regulation of the apoptotic pathway. miRNAs control apoptosis either directly inhibiting expression and translation of proapoptotic molecules or indirectly through regulating the machineries of apoptotic signaling pathways.

Several miRNAs serve as protective factor of cardiomyocyte survival by suppressing/inhibiting the expression of proapoptotic molecules. miR-145 acts on mitochondrial apoptotic pathway by blocking the expression of Bnip3, a mitochondrial apoptosis initiation factor Bnip3. By inhibiting Bnip3 mediated signaling, miR-145 attenuates oxidative stress induced cardiomyocyte cell death caused by H_2_O_2_ or ischemia reperfusion [72]. Interestingly, miR-494 plays vital role in protection against I/R injury induced cell death by activating Akt-mitochondrial signaling pathway and antiapoptotic proteins (FGFR2 and LIF) through inhibition of expression of proapoptotic proteins (PTEN, ROCK1, and CaMKII*δ*) [73]. miR-499 also inhibits mitochondrial pathway of cell death by abolishing calcineurin-mediated dephosphorylation of dynamin-related protein-1 (Drp1) and reducing mitochondrial fission [74]. Another research group found that miR-30 is required to prevent abnormal mitochondrial fission associated cell death. However, the downregulation of miR-30 under oxidative stress positively modulates the expression of p53 and Drp1, which consequently leads to cell death [75]. Surprisingly, miR-30 family can target multiple genes of *β*1 and *β*2 adrenergic receptors and G protein *α* i subunit (Gi*α*2) to regulate *β*-adrenergic signaling pathway [76]. The mitochondrial morphology associated cell death is regulated by miR-181c, which is inversely correlated with the levels of caspases, Bcl-2, and cytochrome C [77]. In diabetic cardiomyopathy, miR-675 can prevent cardiac apoptosis by suppressing the expression of mitochondrial voltage dependent anion channel 1 (VDAC1), which plays crucial role in activation and release of proapoptotic molecules [78].

Under reticulum stress-associated apoptotic process, the upregulation of miR-185 protects cardiac cells from death by targeting Na^+^/H^+^ exchanger-1 (Nhe1) [79]. The upregulation of miR-378 enhances cell survival by inhibiting caspase-3 expression in cardiomyocytes during ischemia reperfusion injury [80]. MiR-210 also promotes cardiac cell survival by blocking expression of the negative regulators of VEGF signaling (Efna3 and Ptp1b) and apoptosis inducing factor-mitochondrion associated 3 (AIFM3) [81, 82]. Similarly, miR-21 [83] and miR-328 [84] directly target programmed cell death 4 (PDCD4) gene and Atp2a2, respectively. Bim is a Bcl2 family protein, which positively regulates apoptosis by activating caspase cascade of death. Bim expression is inhibited by miR-24 [85] and upregulation of miR-24 decreases apoptosis and necrosis in ischemic myocytes by inhibiting BCL2L11 gene [86]. Interestingly, miR-214 prevents Ca^2+^ overload induced cell death by attenuating several Ca^2+^ signaling molecules including NCX1, CaMKII*δ*, CypD, and Bim [87]. In sepsis induced cardiac dysfunction, miR-124 can block cardiomyocyte apoptosis by downregulating the expression of syntaxin-2. However, its expression is suppressed by bacterial LPS under experimental condition [88].

The reduction of blood flow to myocardial tissue produces hypoxia, which imposes oxygen tension and cardiomyocyte cell death. Several miRNAs target hypoxia inducible genes to attenuate or aggravate the oxygen tension induced cardiomyocyte death. miR-199a acts as a master regulator of hypoxia by inhibiting hypoxia inducible factor-1*α* (Hif-1*α*) expression and reducing oxygen tension. MiR-199a indirectly abolishes increased level of Hif-1*α* by blocking the expression of Sirt1, which is required downregulation of prolyl hydroxylase 2, a molecule involved in stabilization of Hif-1*α* [89]. miR-363 controls hypoxia inducible cardiac apoptosis via regulation of Notch1 expression and its downstream signaling [90]. Similarly, the hypoxia induced early expression of miR-146b is cardiomyocyte protective by increasing the expression of NF-kB and STAT3, which play cell survival role [91]. miR-214 protects cardiomyocyte cell survival and tissue damage caused by hypoxia and I/R injury by suppressing PTEN expression activating PI3K-Akt mediated survival signaling [92]. In contrast, the hypoxia inducible expression of miR-200c, miR-92a, and miR-27a promotes cardiomyocyte cell death by targeting GATA-4, Smad 7, and interleukin 10 (IL-10) pathway, respectively [93–95]. GATA4 is a transcription factor required for the cardiomyocyte growth and survival and downregulation of miR-200c promotes GATA-4 dependent expression of antiapoptotic genes such as Bcl2 [93], while inhibition of hypoxia induced increase of miR-92a promotes translation of SMAD7 and blocking NF-kB p65 signaling [94]. miR-138 inhibits expression of Lcn2, a proapoptotic gene by abrogating MLK3/JNK/c-jun signaling pathway [96, 97].

**Figure 4:**
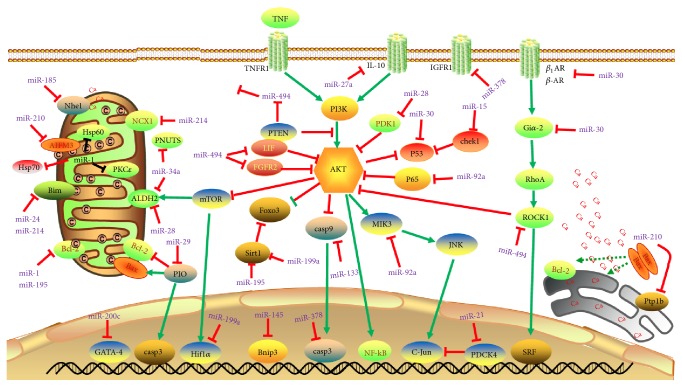
*MicroRNAs (miRNAs) in cardiac apoptosis pathways*. Arrows colored in red indicate the functions of depression; arrows colored in green indicate the functions of activation. Abbreviations for mRNAs: AIFM3: apoptosis inducing factor; Akt: protein kinase B; ALDH2: aldehyde dehydrogenase 2; Bcl-2: B-cell CLL/lymphoma 2; Bim: Bcl2 like 11; Bnip3: Bcl2/adenovirus E1B 19 kDa interacting protein 3; *β*1AR: adrenoceptor *β* 1; *β*2AR: adrenoceptor *β* 2; CaMKII*δ*: calcium/calmodulin-dependent protein kinase II, *δ*; Chek1: checkpoint kinase 1; CypD: Cyclophilin+D; FGFR2: fibroblast growth factor receptor 2; Foxo3: Forkhead box O3; Gi*α*2: G protein *α* i subunit; Hif-1*α*: hypoxia inducible factor 1, *α* subunit; Hsp60: heat shock protein 60; HSP70: heat shock protein 70; IGF-1R: insulin-like growth factor 1 receptor; IL-10: interleukin 10; mTOR: mechanistic target of rapamycin; NCX1: sodium/calcium exchanger 1; Nhe1: Na^+^/H^+^ exchanger 1; PDCD4: programmed cell death 4; PDK1: 3-phosphoinositide-dependent protein kinase-1; PKC*ε*: protein kinase c beta1; PTEN: phosphatase and tensin homolog; Ptp1b: Protein Tyrosine Phosphatase-1B; P53: tumor protein p53; p65: tumor protein p65; Rho-A: Ras homolog family member A; ROCK1: Rho associated coiled-coil containing protein kinase 1; SIRT1: Sirtuin 1; TNFR1: tumor necrosis factor receptor superfamily member 1; TNF: tumor necrosis factor; JNK: c-Jun N-terminal kinase; SRF: serum response factor.

Many miRNAs are upregulated under various pathological conditions in cardiac tissue including myocardial infarction and ischemia/reperfusion injury. They trigger the expression/activity of proapoptotic molecules and cause cardiomyocyte apoptosis. In animal model of ischemia/reperfusion injury, the upregulation of miR-29 inhibits Mcl-2 and enhances Caspase 3 and Bax activity. Interestingly, the activation of peroxisome proliferator-activated receptor (PPAR)-*γ* with pioglitazone abrogates proapoptotic effects of miR-29 and protects cardiac cells from death [98]. miR-122 promotes cardiac apoptosis by inhibiting blockers of caspase-8 and enhancing its expression [99]. Apart from this, miR-378 promotes cardiomyocyte apoptosis by blocking a survival signaling cascade activated by Akt pathway through inhibition of expression of insulin-like growth factor 1 receptor (IGF1R) [100]. Similarly, miR-28 negatively regulates phosphoinositide-dependent kinase-1 (PDK1) and thereby blocks PDK1/Akt/mTOR-dependent signaling of cell survival under oxidative stress condition [101], whereas miR-1 and miR-181c target Bcl-2 expression and favour apoptotic signaling in cardiomyocytes [75, 77]. In addition, overexpression of miR-1 inhibits the expression of protein kinase C*ε* (PKC*ε*) and heat shock protein 60 (HSP60) and that leads to increased expression of caspase-3 in cardiomyocytes under I/R injury [102]. MiR-195 also downregulates Bcl-2 and Sirt1, which subsequently leads to overproduction of ROS to induce cell death in cardiomyocytes [103]. MiR-15 family members play important role in both cardiomyocyte cell proliferation as well as survival. The expression of miR-15 family inhibits a number of cell cycle genes including checkpoint kinase 1 (Chek1) [104], which causes cell cycle arrest and subsequently cell death. miR-28 [105] and miR-34a [106] block the expression of mitochondrial enzyme aldehyde dehydrogenase 2 (ALDH2), which activates activating AMPK and Akt-mTOR signaling and protecting cardiomyocytes against I/R injury. The inhibition of this pathway aggravates the ischemic injury in myocardial cells [105]. The age related increase of miR-34a aggravates apoptotic cell death in the heart with myocardial injury by suppressing PNUTS and DNA damage responses. This leads to functional decline of the heart [107]. The known miRNAs in cardiac apoptosis and their target genes are summarized in Figure 4 and Table 3.

### 4.2. miRNA and Autophagy

**Figure 5:**
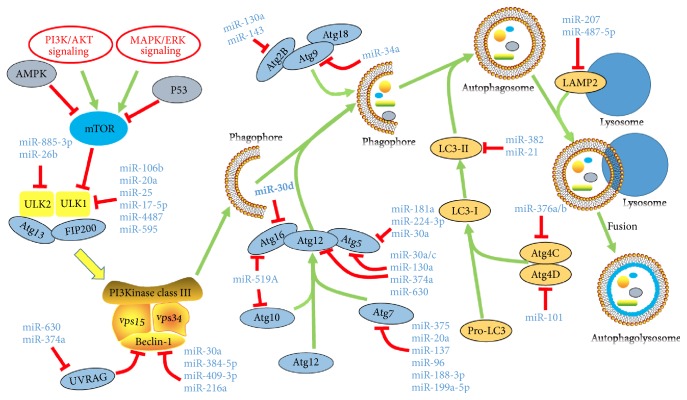
*miRNAs in cardiac Autophagy pathways*. Abbreviations for mRNAs: AMPK: adenosine 5′-monophosphate- (AMP-) activated protein kinase; ATG: AuTophaGy; UVRAG: UV radiation resistance associated gene; VSP: venom serine protease; LAMP2: lysosomal associated membrane protein 2.

Autophagy is an evolutionary conserved and highly regulated cellular recycling program that eliminates damaged intracellular organelles as well as injured cells. Emerging studies indicate that many miRNAs affect autophagy by influencing the expression/translation of different machineries involved in the autophagic process (Figure 5). In mammalian system, the autophagy process is initiated by activation of ULKl or ULK2 complexes. The miRNA-17 family members, miRNA-106b and miRNA-20a, block autophagy process by inhibiting the translation of ULKl by targeting the 3′-UTR of ULKl [108]. Experimental studies have shown that miR-25 [109], miR-17-5p [110], miR-4487, and miR-595 [17] can directly target ULK1, while miR-885-3p [111] and miR-26b [112] block autophagy initiation by inhibiting ULK2 [111]. Following the initiation, the vesicle nucleation is one the key processes in autophagy, in which phosphatidylinositol 3-kinase (PI3K) dependent activation and formation of complex composed of VPS15, VPS34, VPS30, and ATG6/BECN1 (Beclin 1) proteins play a central role in vesicle formation. Several miRNAs can modulate the expression of components of above complex and regulate the vesicle nucleation process. miR-30a, miR-384-5p, miR-409-3p, and miR-216a directly target the expression of Beclin-1 gene to inhibit vesicle formation [113–117].

The process of vesicle elongation is regulated by two ubiquitin-like protein conjugation systems: Atg5-Atg12-Atg16 protein complex conjugation system and LC3 (microtubule-associated protein 1 light chain 3) conjugation system. Several miRNAs regulate autophagic process by modulating the expression of components of these two ubiquitin-like protein system. Jing et al. found that miRNA-30a/c, miR-181a, miR-130a, miR-374a, and miR-630 inhibit the progression of autophagic process by inhibiting expression of Atg5 and Atg12 [191], whereas miR-181a [119], miR-30a [121], and miR-224-3p [120] only interact with Atg5 mRNA resulting in reduction of its translation. In addition, miR-30d directly targets Atg16 to block the formation of Atg5-Atg12-Atg16 protein complex [122]. miR-519A has the capability to suppress expression of both Atg16 and Atg10 [118]. Several research groups found that miR-375, miR-20a, miR-137, miR-96, miR-188-3p, and miR-199a-5p block autophagic process by directly targeting Atg7, an autophagic protein involved in the formation of Atg7-Atg10 complex [32, 123–127]. Atg4 family is involved in the processing of LC3. miR-376 family members such as miR-376a and miR-376b inhibit translation of Atg4C and attenuate autophagic process [128, 129]. miR-101 suppresses expression of Atg4D abd blocks Lc3 processing [130]. Wang et al. [131] found that silencing of endogenous miR-382 function in renal mesangial cells can induce LC3-related autophagy. Ursolic acid is shown to improve autophagy and increase the expression of LC3-II to attenuate diabetic mesangial cell injury through attenuated miRNA-21/PTEN/Akt/mTOR signal pathway [132].

Apart from them, MiR-130a and miR-143 interfere the formation of Atg9-Atg2-Atg18 complex by targeting Atg2B and inhibiting autophagosome assembly [133, 134]. Another research group found that miRNA-34a attenuates autophagic process by downregulating the expression of Atg9 gene [135]. The fusion of autophagosome–lysosome fusion is mediated by key autophagic proteins such as RAB1B, RAB22A, RAB14, RAB27A, LAMP2, and LAMP3. Many research groups found that miR-502 and miR-451 inhibit RAB1B and RAB14, respectively [136, 137]. LAMP2 can be suppressed by miR-207 and miR-487-5p, while miR-205 blocks RAB27A and LAMP3 and interfering autophagic vesicle maturation process [138, 140, 192]. The two other miRNAs such as miR-630 and miR-374a can directly target UVRAG and thus affect the maturation of autophagosomes [118]. The known miRNAs in autophagy and their target genes are illustrated in Figure 5 and Table 4.

### 4.3. miRNA, Inflammatory Response, and Pyroptosis

The activation and accumulation of immune cells such as macrophage are of the earliest responses to cardiac tissue injury that governs the clearance of cell debris, stimulation of compensatory growth and cardiac tissue regeneration. The macrophage polarization and activation are the key process involved in the determination of the inflammatory response, cardiac tissue injury, angiogenesis, scar formation, and cardiac tissue remodeling [139]. In general, the activation of classical M1 macrophage at early phase initiates inflammatory response by increased production of proinflammatory cytokines and chemokines. However, the polarization of macrophages generates alternative M2 macrophage phenotype, which exerts inflammation suppression, apoptotic cell clearance, and tissue repair [139]. miRNAs modulate the activity of macrophages and they can regulate the expression of several proinflammatory cytokines. miR-155 plays multiple roles in the inflammatory process. miR-155 regulates macrophage polarization by controlling SOCS1 and Akt1 axis [141]. In viral myocarditis, the increased expression of miR-155 blocks macrophage polarization and alternatively activated M2 macrophages phenotype transformation. This results in increased pathogen induced inflammation and cardiac injury [142]. miR-155 also promotes macrophage survival by upregulating the SHIP1-Akt signaling cascade [143]. The increased level of macrophage derived miR-155 acts as paracrine regulator of cardiac fibroblast proliferation as well as inflammatory response following the myocardial infarction induced injury [144]. In contrast, another research group found that miR-155 functions as a negative feedback regulator of immune response by reducing the expression of cytokines by cardiac myocytes during viral infection induced myocarditis [145]. Similarly, miR-155 can block the activity of macrophages by directly targeting proinflammatory NF-*κ*B signaling transcription factor in atherosclerotic plaque [146]. Apart from this, the colony stimulating factor 1 (CSF-1) dependent expression of miR-21 positively regulates macrophage polarization. It promotes amplification of M2 macrophage phenotype and directly targets proinflammatory molecules [149]. PPAR*γ* promotes alternative activation and polarization of macrophage (M2) by enhancing the expression of miR-22, which targets Rasa1 and Nfat5 [150]. However, miR-27a switches on M1 macrophage polarization by blocking PPAR*γ* [151]. mir-375 promotes inflammation induced cardiomyocyte cell death by inhibiting PDK1-Akt signaling and increasing proinflammatory cytokines [152].

miR-125 family is considered as a therapeutic target for the prevention of inflammation. miR-125a and miR-125b directly suppress the expression of tumor necrosis factor alpha-induced protein 3 (TNFAIP3, A20), which is a negative regulator of NF-KB signaling [147]. Another research group found that TLR2 and TLR4 dependent upregulation of miR-125a-5p suppresses classical pathway of macrophage activation and inflammation. However, this miR-125a-5p stimulates alternative pathways of macrophage polarization and activation [148]. Interestingly, miR-125b has been reported to suppress the activity and stability of TNF-*α* transcript and thereby it reduces inflammatory responses [123]. Efferocytosis is a process of the clearance of apoptotic cardiomyocyte by macrophages. This process is vital for clear resolution of inflammatory response and tissue repair after cardiac injury. miR-126 promotes the efferocytosis in myocardial tissue. However, the reduction of its expression under diabetic condition impairs the clearance of dead cardiomyocytes, which consequently leads to prolonged inflammation process [153]. Similarly, miR-145-5p can inhibit the CD40 mediated inflammatory response and cardiomyocyte cell death caused by acute hypoxia. But its expression is downregulated under severe hypoxia condition [154]. In sepsis induced cardiac injury and dysfunction, miR-146a can attenuate NF-kB dependent inflammatory signaling by targeting IRAK and TRAF6 in cardiomyocytes and monocytes [155].

Pyroptosis is a proinflammatory form of cell death, which morphologically resembles apoptotic and necrotic mode of cell death. This mode of cell death is triggered by the activation of inflammasome, which leads to the release of inflammatory cytokines. Caspase-1 is the major effector molecule involved in this form of cell death [193]. miR-30d promotes cardiomyocyte pyroptosis under diabetic cardiomyopathy by repressing Foxo3a and its downstream molecule apoptosis repressor with caspase recruitment domain (ARC), which consequently leads to upregulation of the expression of caspase 1 and proinflammatory cytokines (IL-1 and IL-18) in diabetic cardiomyopathy [64]. In opposite to this, miR-9 inhibits inflammatory cytokine secretion and TNF*α* induced pyroptosis by inhibiting the expression of ELAVlike protein 1 (ELAVL1) and this inhibition reduces the expression of NLRP3, caspase-1, and IL-1*β* [194]. Bauernfeind et al. found that miR-223 negatively regulates NLRP3 inflammasome activity and this inhibition could block the initiation of pyroptosis [195]. Currently, very few reports are available to directly link miRNAs and pyroptosis in cardiac tissue. However, their role in the immune cell activation and inflammatory cytokine expression indicates that more miR would be involved in the regulation of pyroptosis process. The known miRNAs in inflammatory response and their target genes are summarized in Table 5.

## 5. miRNA and Angiogenesis

Neovascularization is the process of new blood vessel formation, which is generated from vascular endothelial cells (EC). This process is essential for the blood supply and nutrients to the recovered part of tissues after various injury [196]. The vascular regeneration is one of the important pathological features of ventricular remodeling. In pressure overload myocardial hypertrophy, the growth rate of vascular tissue is less than the processes of cardiomyocyte hypertrophy. This leads to a significant decrease in the density and causes formation of thin myocardial blood vessels, which relatively reduces blood supply to the heart and eventually affecting heart function. Emerging evidences reveal that miRNAs play fundamental role in maintaining the vascular integrity, endothelial cell proliferation, migration, blood vessel formation and sprouting in the adult heart in response to injury. Under hypoxia and vascular injury condition, the upregulation of miR-126 activates endothelial cells (EC) and endothelial progenitor cells (EPCs) in order to promote vascular healing and vessel formation. The endothelial specific expression of miR-126 promotes the development and maintenance of vessels by enhancing the expression of angiogenic factors such as VEGF and FGF. This is achieved by directly suppressing Spred-1and phosphoinositol-3 kinase regulatory subunit 2 (PIK3R2/p85-b), which is a intracellular antiangiogenic signaling [156]. MiR-130a contributes to angiogenesis by suppressing antiangiogenic homeobox gene GAX and HOXA5 in endothelial cells. In addition, miR-130a activates PI3K/Akt dependent signaling by inhibiting PTEN expression and that attenuates remodeling after myocardial infarction induced injury [158, 159]. The miR-17-92 cluster is highly expressed in human endothelial cells. This cluster expresses six miRNAs (miR-17, miR-18a, miR-19a, miR-20a, miR-19b-1, and miR-92a-1), which are transcriptionally regulated by c-Myc [160]. The miRNAs such as miR-494 and miR-126-5p act as proangiogenic factors by targeting bone morphogenetic protein 4 (BMP4) [197], while miR-18a downregulates CTGF expression in response to Myc activation and promotes angiogenesis [198, 199]. MR-19a primarily targets angiogenesis-inhibitor, thrombospondin-1 (Tsp1) in vascular system. Likewise, the hypoxia induced upregulation of miR-210 promotes endothelial cell migration and capillary like structure formation by targeting Receptor Tyrosine Kinase Ligand Ephrin-A3 and PTP1b [81, 161, 162]. miR-126 also contributes to vascular angiogenesis through targeting Cysteine-rich 61 (CCN1) in PTEN/Akt pathway [157]. miR-146a promotes angiogenesis by increasing the expression of VEGF and reducing the fibrotic process at injury site [65].

The growth factors upregulate miR-296 and this upregulation favours angiogenesis by reducing levels of hepatocyte growth factor-regulated tyrosine kinase substrate (HGS) through targeting HGS mRNA, which is responsible for degradation of angiogenic receptors such as VEGFR2 and PDGFRb in endothelial cells [163]. Sonic hedgehog (Shh) signaling is involved in the formation of large-diameter vessel by inducing expression of angiogenic cytokines, including VEGF and angiopoietin-1 (Ang-1) and Ang-2 (Ang-2). It is well known that suppressor of fused (SuFu) is a negative regulator Shh signaling. The upregulation of miR-378 promotes cell survival, tumor growth, and angiogenesis by suppressing SuFu and Fus-1 expression [164]. In addition, increased level of miR-378 expression stimulates endothelial cell angiogenic activity [165]. In experimental animal model, the endothelial expression of miR-132 displayed angiogenic potential by suppressing endothelial p120RasGAP expression and promoting Ras activation, which is required for the induction of neovascularization [166]. miR-23 and miR-27 promote angiogenesis by inhibiting two antiangiogenic factors such as Sprouty2 and Sema6A proteins [167], while miR-27b expression accelerates angiogenesis by regulating translation of Dll4/Notch axis, PPAR*γ* and its downstream effectors [168]. Some miRNAs play distinct role in MI heart. For instance, miR-24 has displayed that different activities depend on the cell types in cardiac tissue. In the postinfarct heart, miR-24 enhances survival, proliferation, and angiogenic function of EC by targeting GATA2, p21-activated kinase PAK4, and eNOS. However, it has detrimental role on cardiomyocytes and fibroblasts by inducing cell death [169, 170].

Several miRNAs oppose the angiogenic process in endothelial cells. miR-214 prevents cardiac angiogenesis by reducing the expression of X-box binding protein 1 (XBP1); an important transcription factor contributes to endothelial cells proliferation and tube formation [171]. In response to stress, the upregulation of miR-34 family members (miR-34a, miR-34b, and miR-34c) [172], in particular, miR-34a, stimulates endothelial progenitor cell senescence and impedes its angiogenic activity through attenuation of the expression of silent information regulator 1 (SIRT1) [173, 174]. In animal model of MI, silencing of miR-34 family attenuates MI-induced pathological left ventricular remodeling after MI and improves cardiac function by suppressing vascular endothelial growth factors (VEGF), vinculin, protein O-fucosyltransferase 1 (Pofut1), Notch1, and semaphorin 4B (Sema4b) [172]. By targeting the proangiogenesis TGFb-1, miR-29a and miR-101a inhibit TGF*β* pathway in endothelial cells in MI rats [175]. Overexpression of miR-15b-5p suppresses arteriogenesis and angiogenesis in mice by directly targeting protein kinase B-3 (AKT3) [176]. Some miRNAs play distinct role in MI heart. For instance, miR-24 has displayed that different activities depend on the cell types in cardiac tissue. In the postinfarct heart, miR-24 enhances survival, proliferation, and angiogenic function of EC by targeting GATA2, p21-activated kinase PAK4, and eNOS. However, it has detrimental role in cardiomyocytes and fibroblasts by inducing cell death [169, 170].

miR-100 has antiangiogenic function by blocking mTOR signaling in endothelial and vascular smooth muscle cells, and its inhibition promotes the regulation of revascularization [177]. EMT is one of the major mechanisms for the development and progression of fibrosis. It is well known that ZEB1/SIP1 is a suppressor of E-cadherin and upregulation of ZEB1/SIP leads to the loss of epithelial phenotype. Surprisingly, miR-200b and ZEB1/SIP1 have a feedback loop regulation to control epithelial-mesenchymal transition (EMT) and increased expression of miR-200b can suppress ZEB1/SIP1 during EMT [178, 179]. In endothelial cells, miR-200b regulates angiogenic signals by targeting VEGF signaling receptors including VEGFR2, Flt1, KDR, and GATA binding protein 2 (GATA2) [180, 181].

**Figure 6:**
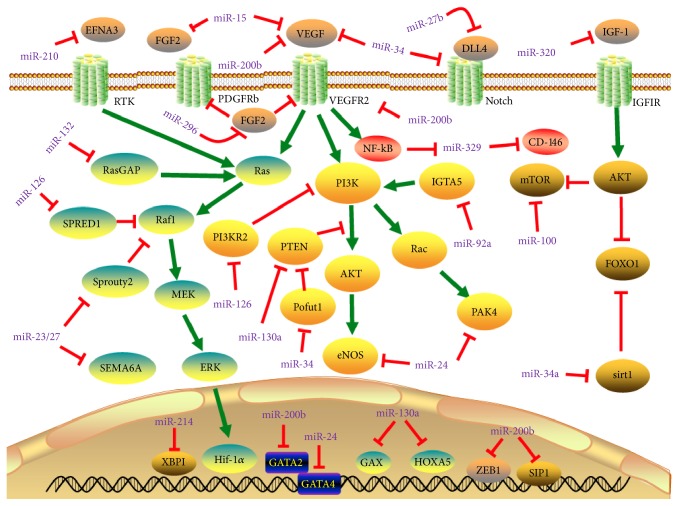
*MicroRNAs (miRNAs) in angiogenesis pathways*. Arrows colored in red indicate the functions of depression; arrows colored in green indicate the functions of activation. Abbreviations for Dll4: delta-like protein 4; Efna3: ephrin A3; eNOS: endothelial nitric oxide synthase; FGF2: fibroblast growth factor 2; Foxo1: Forkhead box O1; GATA2: GATA binding protein 2; Gata4: GATA binding protein 4; GAX: gaseous oxygen; HGS: hepatocyte growth factor-regulated tyrosine kinase substrate; Hif-1*α*: hypoxia inducible factor 1, *α* subunit; HOXA5: homeobox A5; IGF-1: insulin-like growth factor 1; IGF-1R: insulin-like growth factor 1 receptor; mTOR: mechanistic target of rapamycin; PIK3R2: phosphoinositol-3 kinase regulatory subunit 2; PTEN: phosphatase and tensin homolog; RasGAP: Ras GTPase–activating protein; Sema6A: semaphorin 6A; SIP1: Smad interacting protein 1; SIRT1: Sirtuin 1; Sprouty2: sprouty homolog 2; VEGF: vascular endothelial growth factor; VEGFR2: vascular endothelial growth factor receptor 1; XBP1: X-box binding protein 1; ZEB1: zinc finger E-box-binding homeobox 1; RTK: receptor tyrosine kinase; Casp9, caspase 9; Casp3, caspase 3; ERK: extracellular regulated protein kinases; Spread-1: Sprouty-related, EVH1 domain-containing protein 1; PDGFR*β*: platelet-derived growth factor receptor *β*.

Several other miRNAs such as miR-15a, miR-16, and miR-424 target FGF and VEGF signaling components and impose antiangiogenic effects. MiR-15a suppresses FGF2 and VEGF expression, but miR-16 and miR-424 block VEGFR2 and FGFR1 receptor expression in endothelial cells [183, 184]. When the glucose availability is high and growth factor level is low, the endothelial expression of miR-503 is increased, which causes impairment of endothelial function and angiogenesis via directly inhibiting molecules involved in cell proliferation and survival proteins such as cdc25A and CCNE1 [182]. The upregulation of miR-320 expression suppresses insulin-like growth factor-1 (IGF-1) activity in myocardial microvascular endothelial cells and thus impaired angiogenesis in diabetic condition [185]. Astonishingly, the expression of miR-320 by cardiomyocytes controls endothelial function under diabetic condition. In experimental diabetic animals, miR-320 is transferred from cardiomyocytes to endothelial cells by exosomal activity and this transfer regulates endothelial cells migration and tube formation by downregulating IGF-1, Hsp20, and Ets2 [186]. miR-329 abrogates angiogenesis by targeting CD146, an endothelial adhesion molecule required to maintain endothelial cell integrity [187]. In cardiac ischemic condition, miR-92a targets integrin subunits a5 (ITGA5) and causes blockage on angiogenesis, which worsens the ischemic injury [188, 189]. The suppression of miR-92a with antagonist after ischemia reperfusion injury improves the functional recovery of damaged tissue by promoting blood vessel growth [190]. The known miRNAs involved in angiogenesis and their target genes are summarized in Figure 6 and Table 6.

## 6. Conclusion

Myocardial remodeling due to various insults to the heart is a multifactorial and complex process. To prevent and manage the cardiac dysfunction due to postinjury related cardiac remodeling, it is important to have a clear understanding about the occurrence, development, and progression of cardiac remodeling in the injured heart. Numerous research findings in recent years suggest that miRNAs have unavoidable role in cardiac structure remodeling by differentially regulating thousands of different mRNAs. Many miRNAs have been proposed as prognosis factors and therapeutic tools for myocardial remodeling. Thus, miRNA-based strategies can be a promising approach to target network of myocardium remodeling related genes. In recent years, the pharmacological development of miRNA inhibitors/activators such as miR-mimics, antagomiRs, and decoys to manipulate miRNAs expression level has made a significant progress to utilize them as therapeutic tools for cardiac failure caused by myocardial remodeling. In experimental models mimicking human heart diseases, the exogenous administration of miRNA inhibitors or miRNA mimics can alleviate the remodeling process and improve cardiovascular disease. However, specificity, half-life of these molecules, efficiency, and route of administration are the major challenge to bring them from the workbench to the bedside. By solving these issues by joint efforts of basic researchers and clinical scientist, miRNA could be better and most effective therapeutic regimen to manage cardiovascular problems associated with cardiac remodeling.

## Figures and Tables

**Table 1:** Summary of reported miRNAs and their targets in cardiac hypertrophy.

miRNAs	Targets	Effector cells	Signal pathways	References
Antihypertrophic				
miR-1	Eif4e	C57Bl/6 mouse and NMCMs	Translation	[7–12]
	Cdk6	NRVCs and ACC mouse	CDKs-Rb pathway	
	HDAC6	NRCMs and Wistar rats	Chromatin modifying	
	CAM	TG mouse and NRVMs	Calcium signaling	
	Mef2a	TG mouse and NRVMs	Calcium signaling	
	Gata4	TG mouse and NRVMs	Calcium signaling	
	FABP3	NMCMs and TAC mouse	PPAR	
	Fbln2	NMCMs	ECM	

miR-10a	Tbx5	TAC mouse and NRVMs	transcription	[15]

miR-497	Sirt4	TAC mouse and NRVMs	AMPK	[16]

miR-133	AT1R	Male Wistar rats and NRCMs	ERK-MAPK	[13, 14]
	Cdc42,	NMCMs and TAC mouse	MAPK	
	Rho-A,	NMCMs and TAC mouse	cGMP-PKG	
	Nelf-A/WHSC2	NMCMs and TAC mouse	Transcription	

miR-223	TNNI3K	NRCMs and TAC mouse	Calcium signaling	[17]

miR-455	CRT	TAC mouse	Calcium signaling	[18]

miR-378	Mapk1	NRCMs and TAC mouse	MAPK	[19, 20]
	IGF1r	NRCMs and TAC mouse	MAPK	
	Grb2	NRCMs and TAC mouse	MAPK	
	Ksr1	NRCMs and TAC mouse	MAPK	

miR-145	GATA6	NRCMs and TAC mouse	cGMP-PKG	[21, 22]

miR-29a-3p	NFATc4	H9c2 cell	cGMP-PKG	[26]

Prohypertrophic				
miR-21-3p	SORBS2	NRVMs and NRCFs	Transcription	
	PDZ	NRVMs and NRCFs	Transcription	[32]
	PDLIM5	NRVMs and NRCFs	Transcription	

miR-208	AT1R	Male wistar rats and NRCMs	ERK-MAPK	[27, 28, 44]
	THRAP1	TG mouse	Thyroid hormone	
	Myh7	Adult male Dahl salt-sensitive rats		

miR-124	Grp78	NRCMs	Reticulum (ER) stress signaling	[29]
	CRT	NRCMs	Calcium signaling	

miR-297	Sig-1R	TAC rat and NRVMs	ER stress signaling	[30]

miR-17-3p	TIMP3	NRVMs and C57BL/6 mice	PTEN-AKT pathway	[31]

miR-155	Mef2A	TAC rat and KO mice	Calcineurin pathway	
	Jarid2	TAC rat and KO mice	Calcineurin pathway	[23, 33]
	AGTR1	Rat H9C2 cell	Calcium signaling pathways	

miR-212/132	Foxo3	H9c2 cells and TAC mouse	PI3K-Akt	[24]

miR-22	Hdac4	NRVCs, miR-22 KO mouse	AMPK	
	Pten	NRVCs	PI3K-AKT	[25, 34, 35]
	Sirt1	NRVCs, miR-22 KO mouse	AMPK	

miR-23a	Foxo3a,	NMCMs, TAC and TG mouse	PI3K-AKT	[25, 36]
	LPA1	NMCMs	PI3K-AKT	

miR-195	HMGA1	NRVCs		[37, 38]

miR-19a/b	atrogin1,	NRVCs and TAC mouse	calcineurin/NFAT	[39]
	Murf1	NRVCs and TAC mouse	PKC	

miR-27b	Ppar*γ*	NRVCs, TAC and TG mouse	PPAR	[40]

miR-328	Serca2a	NRVCs, TAC and TG mouse	cGMP-PKG	[41]

miR-185	Camk2d	NRVMs and TAC mouse	Calcium	[42]

miR-101	Ncx1	NRVMs and TAC mouse	cGMP-PKG	[43]
	Nfatc3	NRVMs and TAC mouse	cGMP-PKG	
	Rab1A	TAC rat	MAPKK	

LPA1: lysophosphatidic acid receptor 1; Mapk1: mitogen-activated protein kinase 1; Murf1: tripartite motif-containing 63; NCX1: sodium/calcium exchanger 1; Nfatc3: nuclear factor of activated T cells, cytoplasmic, calcineurin dependent 3; NFATc4: nuclear factor of activated T cells 4; PDLIM5: PDZ and LIM domain 5; Ppar*γ*: peroxisome proliferator-activated receptor *γ*; p300: E1A binding protein p300; Rab1A: Ras-related protein Rab 1a; RasGAP: Ras GTPase–activating protein; Rheb: Ras homolog enriched in brain; Rho-A: Ras homolog family member A; Serca2a: Sarco/endoplasmic reticulum Ca^2+^-ATPase 2a; SORBS2: SH3 domain-containing protein 2; Sox6: sex-determining region Y box 6; THRAP1: mediator complex subunit 13; TNNI3K: troponin I type 3 interacting kinase; NRVCs: neonatal rat ventricular cardiomyocytes; AAC: abdominal aortic constriction; TBX5: T-box 5; NRCMs: neonatal rat cardiomyocytes; SORBS2: SH3 domain-containing protein 2; PDLIM5: PDZ and LIM domain 5; NRVMs: neonatal rat ventricular myocytes; NRCFs: neonatal rat cardiac fibroblasts.

**Table 2:** Summary of reported miRNAs and their targets in cardiac fibrosis.

miRNAs	Targets	Effector cells	Signal pathways	References
Antifibrosis				
miR-101a	TGF-*β*R1,	NRCFs and MI rat	TGF*β* signaling	[47, 48]
	c-Fos	NRCFs and MI rat	MAPK	

miR-133/30	CTGF	RCMs, RCFs and Ren2 rat	TGF*β* signaling	[59]

miR-24	Furin	MCFs and MI mouse	TGF*β* signaling	[50]

miR-29	Elastin	RCFs and MI mouse	Protein digestion/absorption	[61–63]
	Fbn1	RCFs and MI mouse	ERK	
	Col1*α*1	RCFs and MI mouse	ERK	
	Col1*α*2	RCFs and MI mouse	ERK	
	Col3*α*1,	RCFs and MI mouse	ERK	
	FBN,	RCFs and MI mouse	ERK	

miR-146a	VEGF	hMSCs	VEGF	[65]

miR-98	TGF*β*R1	HCFs	TGF*β*	[51]

miR-433	AZIN1		TGF*β*	[67]
	JNK1		TGF*β*	

miR-142-3p	HMGB1	M6200 cells	TGF-*β*1/Smad3 signaling pathway	[66]

miR-29b	Tgf*β*1,	MCFs	TGF*β*	[64]

miR-19a-3p/19b-3p	TGF*β* RII	HCFs	TGF*β* signaling	[52]

miR-22	Smad4	MCFs	TGF-*β*-pathway	[58]

miR-378	TGF-*β*1	TAC mouse NMCMs and NRCFs	TGF/Smad signaling	[55]

miR-26a	COL1,	NRCFs, TAC and miR-26a TG mouse	PI3K-AKT	[60]
	CTGF	NRCFs, TAC and miR-26a TG mouse	ECM	

miR-15 family	TGF-*β*R1	MI mouse and NMCMs	TGF-*β*-pathway	
	p38	MI mouse and NMCMs	TGF-*β*-pathway	
	SMAD3	MI mouse and NMCMs	TGF-*β*-pathway	[53, 54]
	SMAD7	MI mouse and NMCMs	TGF-*β*-pathway	
	Endoglin	MI mouse and NMCMs	TGF-*β*-pathway	

Profibrosis				
miR-21	Spry1	NRCFs, NRCMs, TAC and TG mous	ERK-MAPK	[56, 57]
	TGF*β*RIII	MI mouse and NRCFs	TGF*β*1-Smad3 signaling	

miR-208a	Endoglin	TAC mouse and RCFs	TGF-*β*-pathway	[28, 68, 69]
	b-MHC	TAC mouse and RCFs	TGF-*β*-pathway	
	THRAP1	TG mouse	Thyroid hormone	

miR-499	Akt	NRCFs	Akt	[68]
	MAPKs	NRCFs	MAPK	
	Egr1	NRCFs	ERK	
	Egr2	NRCFs	ERK	
	Fos	NRCFs	MAPK	
	Myh7	NRCFs	ERK	
	Acta1	NRCFs	Smads	

miR-122	TGF-*β*1	HCFs	TGF*β*	[49]

miR-125b	Apelin	HCFs, TAC and Ang II induced mouse	TGF*β*	[70, 71]

Akt: protein kinase B; *β*-MHC: beta myosin heavy chain; c-Fos: proto-oncogene protein; COL1: collagen, type 1; Col1*α*2: collagen, type 1 *α* 2; Col3*α*1: collagen, type 3 *α* 1; CTGF: connective tissue growth factor; Egr1: early growth response protein 1; Egr2: early growth response protein 2; FBN: fibrillin; Fbn1: fibrillin 1; MAPKs: mitogen-activated protein kinases; p38: tumor protein p38; SMAD3: SMAD family member 3; SMAD2: SMAD family member 2; SMAD7: SMAD family member 7; Spry1: sprouty homolog 1; TGF*β*: transforming growth factor *β*; TGF-*β*1: transforming growth factor *β* 1; TGF-*β*R1: transforming growth factor *β* receptor 1; TGF*β*RIII: transforming growth factor *β* receptor III; THRAP1: mediator complex subunit 13; RCFs: rat cardiac fibroblasts; MCFs: mouse cardiac fibroblasts; HCF: human cardiac fibroblasts; hMSCs: human mesenchymal stem cells; HBMVECs: human brain microvascular endothelial cells.

**Table 3:** Summary of reported miRNAs and their targets in cardiac apoptosis.

miRNAs	Targets	Effector cells	Signal pathways	References
Antiapoptosis				
miR-145	Bnip3	I/R mouse	FOXO	[72]

miR-199a	Hif-1*α*, Sirt1	NRCMs NRCMs	mTOR AT1R	[89]

miR-21	PDCD4	NRCMs	NF-kB	[83]

miR-328	Atp2a2	H9C2 cells		[84]

miR-214	NCX1 PTEN	NRCM and miR-214 KO mouse I/R mouse	Calcium signaling PI3K-AKT	[87] [92]

miR-24	Bim	NMCMs	Mitochondrial apoptosis	[85, 86]

miR-146b	RNase L	H9c2 cells	NF-kB	[91]

miR-378	Caspase-3	H9c2 cells and AMI rat	MAPK	[80]

miR-494	PTEN ROCK1 CaMKII*δ* FGFR2 LIF	miR-494 TG Mouse miR-494 TG Mouse miR-494 TG Mouse miR-494 TG Mouse miR-494 TG Mouse	PI3K-AKT cGMP-PKG HIF1 PI3K-AKT TNF	[73]

miR-499	Drp1	Male C57BL/6 mice	Mitochondrial pathway	[74]

miR-185	Nhe1	NRVMs	cAMP	[79]

miR-30 family	*β*1AR, *β*2AR, Gi*α*2, P53 Drp1	MI rat, DOX-induced HF rat, ARCM and H9c2 cells MI rat, DOX-induced HF rat, ARCM and H9c2 cells MI rat, DOX-induced HF rat, ARCM and H9c2 cells NRCMs NRCMs	*β*-adrenergic pathway *β*-adrenergic pathway *β*-adrenergic pathway p53 mitochondrial pathway	[75, 76]

miR-210	Efna3, Ptp1b AIFM3	Mouse HL-1 cardiomyocytes and adult FVB mice Mouse HL-1 cardiomyocytes, adult FVB mice H9c2 cells,neonatal rat cardiomyocytes (NRCM)	VEGF signaling VEGF signaling Mitochondrial apoptosis	[81, 82]

miR-92a	NF-kB p65	rat H9c2 cells	NF-kB	[94]

miR-675	VDAC1	DCM	Mitochondrial apoptosis	[78]

miR-138	Lcn2	HL-1 cells	Mitochondrial apoptosis	[96, 97]

miR-124	STX2	Male BALB/c rats	miR-124a/STX2 pathway	[88]

Proapoptosis				
miR-1	HSP60 Bcl-2 PKC*ε*	LNA-antimiR-1 treated mouse H9c2 cells and I/R rat LNA-antimiR-1 treated mouse	RNA degradation Mitochondrial apoptosis cGMP-PKG	[75, 102]

miR-200c	GATA-4	NMCMs	cGMP-PKG	[93]

miR-363	Notch1	Rat H9C2 cell	Notch signaling	[90]

miR-122	caspase-8	NMCMs	ERK-MAPK	[99]

miR-181c	Bcl-2	NMCMs	Mitochondrial apoptosis	[77]

miR-15	Chek1	C57BL/6 mice and TG mouse		[104]

miR-34a	PNUTS ALDH2	NRCMs and MI rat NRCMs and MI rat	Oxidative stress Oxidative stress	[106, 107]

miR-378	IGF1R	NMCMs and H9C2 cells	MAPK	[100]

miR-27a	IL-10	Sprague-Dawley rats and H9c2 cell	Interleukin 10 (IL-10) pathway	[95]

miR-29	PIO	H9c2 cells	PPAR	[98]

miR-28	PDK1 ALDH2	NMCMs NMCMs	PDK1/Akt/mTOR-dependent signaling AMPK and Akt-mTOR signaling.	[101, 105]

miR-195	Sirt1 Bcl-2	C57BL/6 mouse and NMCMs C57BL/6 mouse and NMCMs	AMPK Mitochondrial apoptosis	[103]

AIFM3: apoptosis inducing factor; ALDH2: aldehyde dehydrogenase 2; Bcl-2: B-cell CLL/lymphoma 2; Bim: Bcl2 like 11; Bnip3: Bcl2/adenovirus E1B 19 kDa interacting protein 3; *β*1AR: adrenoceptor *β* 1; *β*2AR: adrenoceptor *β* 2; CaMKII*δ*: calcium/calmodulin-dependent protein kinase II, *δ*; Chek1: checkpoint kinase 1; CypD: Cyclophilin+D; Drp1: dynamin-related protein-1; Efna3: ephrin A3; FGFR1: fibroblast growth factor receptor 1; Gi*α*2: G protein *α* i subunit; Hif-1*α*: hypoxia-inducible factor 1, *α* subunit; HSP60: heat shock protein 60; IGF-1R: insulin-like growth factor 1 receptor; IL-10: interleukin 10; Lcn2: lipocalin-2; LIF: leukemia inhibitory factor; NCX1: sodium/calcium exchanger 1; Nhe1: Na+/H+ exchanger 1; PDCD4: programmed cell death 4; PDK1: 3-phosphoinositide-dependent protein kinase-1; PKC*ε*: protein kinase c beta1; PTEN: phosphatase and tensin homolog; Ptp1b: Protein Tyrosine Phosphatase-1B; P53: tumor protein p53; p65: tumor protein p65; ROCK1: Rho associated coiled-coil containing protein kinase 1; SIRT1: Sirtuin 1; SMAD7: SMAD family member 7; DCM: diabetic cardiomyopathy.

**Table 4:** Summary of reported miRNAs and their targets in Autophagy.

miRNAs	Targets	Effector cells (Tissue)	References
*miRNAs regulate the induction of autophagy*			
miR-106b	ULKl	C2C12 (myoblast cells)	[108]
miR-25	ULKl	MCF-7 (breast cancer)	[109]
miR-17-5p	ULKl	TIB-71 (murine macrophage cells)	[110]
miR-4487	ULKl	SH-SY5Y (neuroblastoma)	[17]
miR-595	ULKl	SH-SY5Y (neuroblastoma)	[17]
miR-885-3p	ULK2	JHU-029 (squamous cell carcinoma)	[111]
miR-26b	ULK2	PC3, C4-2 (prostate cancer)	[112]

*miRNAs regulate the vesicle nucleation of autophagy*			
miR-30a	Beclin-1	786-0, A489 (renal carcinoma), MG-63 (osteosarcoma)	[113, 114]
miR-384-5p	Beclin-1	primary mouse macrophages	[115]
miR-409-3p	Beclin-1	Lovo Oxa R (colorectal cancer)	[116]
miR -216a	Beclin-1	PANC-1 (pancreas cancer)	[117]
miR-630	UVRAG	JHU-029 (squamous cell carcinoma)	[118]
miR-374a	UVRAG	JHU-029 (squamous cell carcinoma)	[118]

*miRNAs regulate the vesicle elongation and retrieval of autophagy*			
miR-181a	Atg5	MCF-7 (breast cancer) Huh7 (liver cancer) K562 (chronic myelogenous leukemia)	[119]
miR-224-3p	Atg5	U251 and U87 (glioblastoma)	[120]
miR-30a	Atg5	K562 (CML)	[121]
miR-30d	Atg16	A2780, OVCAR10 and 2008 (ovarian cancer), T47D and MCF-7 (breast cancer)	[122]
miR-375	ATG7	Huh7, Hep3B (liver cancer)	[123]
miR-20a	ATG7	SiHa (cervical cancer)	[124]
miR-137	ATG7	U87 (glioblastoma)	[125]
miR-96	ATG7	LNCaP, 22Rv1, and LAPC4 (prostate cancer)	[32]
miR-188-3p	ATG7	NMCMs	[126]
miR-199a-5p	ATG7	Huh7, HepG2 (liver cancer)	[127]
miR-519A	ATG16, ATG10	JHU-029 (squamous cell carcinoma)	[118]
miR-376a/b	ATG4C	MCF-7 (breast cancer) Huh7 (liver cancer)	[128, 129]
miR-101	ATG4D	MCF-7 (breast cancer)	[130]
miR-382	LC3	renal mesangial cells	[131]
miR-21	LC3-II	diabetic mesangial cell	[132]
MiR-130a	Atg2B	MEC-1 (leukemia)	[133]
miR-143	Atg2B	H1299 (lung cancer)	[134]
miR-34a	Atg9	NMCMs	[135]

*miRNAs regulate the formation and degradation of autolysosome*			
miR-502	RAB1B	HCT116 (colorectal cancer)	[136]
miR-451	RAB14	A549, SPC-A1, and NCI-H520 (lung cancer)	[137]
miR-207	LAMP2	primary cortical neuronal cells	[138]
miR-487-5p	LAMP2	A549, H1299 (lung cancer)	[139]
miR-205	RAB27A, LAMP3	DU145, PC3 (prostate cancer)	[140]

AMPK: adenosine 5′-monophosphate- (AMP-) activated protein kinase; mTOR: mammalian target of rapamycin; ATG: AuTophaGy; UVRAG: UV radiation resistance associated gene; VSP: venom serine protease; LAMP2: lysosomal associated membrane protein 2; NMCMs: neonatal mouse cardiomyocytes; RAB1B: member RAS oncogene family; RAB14: member RAS oncogene family; RAB27A: member RAS oncogene family; LAMP3: lysosomal associated membrane protein 3.

**Table 5:** Summary of reported miRNAs and their targets in Inflammatory Response.

miRNAs	Targets	Regulation factors	References
MiR-155	SOCS1, SHIP1, IL-13Ra1	IFN-b, TNF-a, IL-1	[141–146]
miR-125a	TNFAIP3 (A20)	LPS	[147]
miR-125b	TNFAIP3, TNF-a	LPS	[123, 147]
miR-125a-5p	TLR2, TLR4	LPS	[148]
miR-21	CSF-1R	LPS	[149]
miR-22	Rasa1, Nfat5	PPARg	[150]
miR-27a	PPARg	M-CSF	[151]
Mir-375	PDK1	M-CSF	[152]
MiR-126	ADAM9	LPS	[153]
MiR-145-5p	CD40	IL-1*β*, TNF-*α*, IL-6	[154]
MiR-146a	IRAK, TRAF6	LPS, TNF-*α*	[155]

SOCS1: suppressor of cytokine signaling 1; SHIP1: inositol polyphosphate-5-phosphatase D; TNF-a: tumor necrosis factor a; LPS: lipopolysaccharides; CSF-1R: colony stimulating factor 1 receptor; Rasa1: RAS p21 protein activator 1; Nfat5: nuclear factor of activated T-cells 5; PDK1: pyruvate dehydrogenase kinase 1; IRAK: interleukin 1 receptor associated kinase; TRAF6: TNF receptor associated factor 6; ADAM9: A disintegrin and metalloproteinase 9.

**Table 6:** Summary of reported miRNAs and their targets in angiogenesis.

miRNAs	Targets	Effector cells	Signal pathways	References
Proangiogenesis				
miR-126	Spred-1	HEPCs	VEGF	
	PIK3R2	HEPCs	VEGF	[156, 157]
	CCN1	HEPCs	TEN/AKT	

miR-130a	GAX	HUVECs and EGM-2	Transcription	
	HOXA5	HUVECs and EGM-2	Transcription	[158, 159]
	PTEN	MI mouse	PI3K/Akt	

miR-17–92 cluster	Tsp1	HUVECs	VEGF	
	CTGF	HUVECs	TGF*β*	[160]
	BMP4	HUVECs	Bmp4/Smad	

miR-210	Ephrin-A3,	HUVECs	VEGF	[81, 161, 162]
	PTP1b	MHL-1Cs	VEGF	

miR-146a	VEGF	hMSCs	Shh	[65]

miR-296	HGS	HBMVECs		[163]

miR-378	SuFu	U87 cells	Shh	[164, 165]
	Fus-1	U87 cells	Shh	

miR-132	p120RasGAP	HUVECs	Ras	[166]

miR-23/27	Sprouty2,	HUVECs	ERK-MAPK	[167]
	Sema6A	HUVECs	VECF	

miR-27b	Dll4,	LLC1 and ATCC	Dll4/Notch	[168]
	PPAR*γ*	LLC1 and ATCC	PPAR	

miR-24	GATA2,	HVSMCs and HUVECs	cGMP-PKG	[169, 170]
	PAK4	HVSMCs and HUVECs	TGF-*β*1/Smads	
	eNOS	MI mouse, HUVECs and HMVECs	PI3K/Akt	

Antiangiogenesis				
miR-214	XBP1	HUVECs	VEGF	[171]

miR-34 family	SIRT1	HUVECs, HMVECs and HAECs	AMPK	[172–174]
	vinculin	LNA-antimiR-34–treated MI mice and H9c2 cell	FAK1	
	Pofut1	LNA-antimiR-34–treated MI mice and H9c2 cell	Notch	
	Notch1	LNA-antimiR-34–treated MI mice and H9c2 cell	Notch	
	VEGF	LNA-antimiR-34–treated MI mice and H9c2 cell	VEGF	
	Sema4b	LNA-antimiR-34–treated MI mice and H9c2 cell	VECF	

miR-29a/101a	TGFb-1	MI rats	TGFb pathway	[175]

miR-15b-5p	AKT3	HUVECs	PI3K-Akt	[176]

miR-100	mTOR	ESMCs, VSMCs	mTOR	[177]

miR-200b	ZEB1/SIP1	MDCKCs and HBCCs	mTOR	[178–181]
	GATA2	Dermal wound-edge ECs	VEGF	
	VEGFR2	Dermal wound-edge ECs	VEGF	
	Flt1	HUVECs	VEGF	
	KDR	HUVECs	VEGF	

miR-503	cdc25A	HUVECs, HMVECs and HVSMCs	MAPK	[182]
	CCNE1	HUVECs, HMVECs and HVSMCs	Notch	

miR-15a	FGF2	HUVECs	PI3K-AKT	[183, 184]
	VEGF	HUVECs	VEGF	

miR-320	IGF-1	MCECs, NRCMs	PI3K-Akt	[185, 186]
	Hsp20	MCECs, NRCMs	RNA degradation	
	Ets2	MCECs, NRCMs	PI3K-Akt	

miR-329	CD146	HUVECs, HMECs	VEGF and TNF-*α*	[187]

miR-92a	ITGA5	RMUG-S, OVISE and RMG-1	Rho/ROCK	[188–190]

CCNE1: cyclin E1; cdc25A: cell division cycle 25A; CTGF: connective tissue growth factor; Dll4: delta-like protein 4; eNOS: endothelial nitric oxide synthase; Ets2: Euro Truck Simulator 2; FGF2: fibroblast growth factor 2; FGFR1: fibroblast growth factor receptor 1; GATA2: GATA binding protein 2; GAX: gaseous oxygen; HGS: hepatocyte growth factor-regulated tyrosine kinase substrate; HOXA5: homeobox A5; IGF-1: insulin-like growth factor 1; ITGA5: integrin subunits a5; mTOR: mechanistic target of rapamycin; PIK3R2: phosphoinositol-3 kinase regulatory subunit 2; Pofut1: protein O-fucosyltransferase 1; Ppar*γ*: peroxisome proliferator-activated receptor *γ*; PTEN: phosphatase and tensin homolog; Sema4b: semaphorin 4B; Sema6A: semaphorin 6A; SIP1: Smad interacting protein 1; SIRT1: Sirtuin 1; Sprouty2: sprouty homolog 2; Tsp1: thrombospondin-1; VEGF: vascular endothelial growth factor; VEGFR2: vascular endothelial growth factor receptor 1; XBP1: X-box binding protein 1; ZEB1: zinc finger E-box-binding homeobox 1; FAK: focal adhesion kinase; HUVECs: human umbilical vein ECs; EGM-2: endothelial growth medium 2; MHL-1Cs: mouse HL-1 cardiomyocytes; hMSCs: human mesenchymal stem cells; Shh: sonic hedgehog signaling; HBMVECs: human brain microvascular endothelial cells; LLC1: Lewis lung carcinoma; ATCC: RAW 264.7 mouse macrophage cell lines; HVSMCs: human vascular smooth muscle cells; HUVECs: human umbilical vein endothelial cells; HMVECs: human microvascular ECs; HAECs: human aortic endothelial cell; ESMCs: endothelial smooth muscle cells; VSMCs: vascular smooth muscle cells; HVSMCs: human VSMCs cells; MDCKCs: Madin-Darby canine kidney cells; HBCCs: human breast cancer cells; dermal wound-edge ECs: dermal wound-edge endothelial cells; MCECs: mouse cardiac endothelial cells; NRCMs: neonatal rat cardiomyocytes; HMECs: human microvascular endothelial cells.
